# Multiple types of programmed necrosis such as necroptosis, pyroptosis, oxytosis/ferroptosis, and parthanatos contribute simultaneously to retinal damage after ischemia–reperfusion

**DOI:** 10.1038/s41598-022-22140-0

**Published:** 2022-10-13

**Authors:** Galina Dvoriantchikova, Karin Rose Lypka, Emily Victoria Adis, Dmitry Ivanov

**Affiliations:** 1 Department of Ophthalmology, Bascom Palmer Eye Institute, University of Miami Miller School of Medicine, 1638 NW 10th Ave, Miami, FL 33136 USA; 2 Department of Microbiology and Immunology, University of Miami Miller School of Medicine, Miami, FL 33136 USA

**Keywords:** Retina, Ocular hypertension, Necroptosis, Cell death and immune response, Cell death in the nervous system

## Abstract

Ischemia–reperfusion (IR) injury is implicated in a large array of pathological conditions in the retina. Increasing experimental evidence suggests that programmed necrosis makes a significant contribution to inflammation and retinal damage triggered by IR. Since there are many types of programmed necrosis, it is important to identify those involved in retinal IR to determine the correct treatment. To this end, we used a mouse model of retinal IR and a variety of approaches including RNA-seq data analysis. Our RNA-seq data revealed the rapid development of ischemic pathology in the retina during the first 24 h after reperfusion. We found that at least four types of programmed necrosis including necroptosis, pyroptosis, oxytosis/ferroptosis, and parthanatos are simultaneously involved in retinal IR. Our data suggest that the high activity of the TNF pathway at the early stage of retinal IR leads to early activation of necroptosis while significant activity of other types of programmed necrosis appears later. Our results indicate that TNF, glutamate, and ferrous iron generated by Steap3 may be key players concurrently triggering at least necroptosis, oxytosis/ferroptosis, and parthanatos in ischemic retinal ganglion cells (RGCs). Thus, multiple signaling cascades involved in programmed necrosis should be synchronously targeted for therapeutic purposes to treat retinal IR.

## Introduction

Ischemia–reperfusion (IR) injury to retinal tissue is a clinical condition that frequently leads to visual impairments and even blindness, affecting patients' quality of life and functional status^1–6^. Retinal ischemia, whether chronic or acute, has been implicated in a myriad of retinal disorders, including ischemic optic neuropathy, glaucoma, diabetic retinopathy, retinopathy of prematurity, etc.^1–6^. But even in this modern age of gene editing and precision immunotherapy, our ability to regulate the pathogenesis of IR-induced retinal injury remains crudely limited to slowing the rate of degenerative change. The development of novel therapies (i.e., to halt or reverse retinal degeneration) will require a far deeper understanding of the precise mechanistic underpinnings of retinal IR injury. This knowledge can provide us with clinically effective treatments for many retinal diseases.

Although the cause of retinal IR injury is multifactorial, increasing experimental evidence suggests a crucial role of sterile inflammation, or the innate immune response in the absence of live pathogens, in the retinal damage triggered by IR^7–13^. But what is the source of sterile inflammation in the IR retina? By eliminating such source or trigger, degenerative conditions can be improved at the root of the problem, rather than simply concealing symptoms with medications. The ultimate consequence of IR injury to retinal tissue is death by apoptosis and necrosis of retinal ganglion cells (RGCs), a small population of retinal neurons capable of sending visual information directly to the visual cortex of the brain^14–16^. However, apoptosis is not as dangerous in the tissue as necrosis. Apoptotic cell death stimulates the production of anti-inflammatory and neuroprotective factors from immune cells that have internalized apoptotic cells^17–22^. Our study indicates that therapeutic strategies based on mimicking a systemic increase in levels of apoptotic signals can significantly reduce IR-induced retinal injury^17^. Thus, apoptosis is bad news for the cell that triggers this signaling cascade, but good news for the cells that surround it. Meanwhile, cell necrosis is bad news for all neighbors since, as a source of sterile inflammation, it triggers a strong cytotoxic pro-inflammatory response in a tissue^13,18,20,23–25^.

We have learned over the past decade that cell death by necrosis, can be either accidental or programmed (or regulated; occurs via signaling cascades)^26–30^. The results of our colleagues and our data allowed us to reconstruct the following chain of events (“damage chain reaction”) taking place in the retina after IR^7–42^. The initial ischemic stress leads to the accidental RGC necrosis (primary injury) and the release of endogenous factors (proteins, RNA, DNA, etc.; known as damage-associated molecular patterns or DAMPs) from necrotic cells. These factors act through pattern recognition receptors (PRRs) such us toll-like receptors (TLRs) located on the surface of glial cells (small populations of retinal cells known as astrocytes and microglia) and infiltrating leukocytes, causing a toxic pro-inflammatory response and leading to RGC death by apoptosis and programmed necrosis. This cycle can be repeated many times and results in significant retinal damage (secondary injury). Only an anti-inflammatory response mediated by apoptotic cell death or complete tissue destruction can stop this vicious cycle, while programmed necrosis, reproducing itself, causes significant secondary injury^7–42^. There are numerous types of programmed necrosis, the significance of which may vary in different tissues^26–30^. Many drugs have already been created that can prevent different types of programmed necrosis in tissues. However, often drugs that are effective for one type of programmed necrosis are ineffective against another type since the signaling cascades that regulate these types of programmed necrosis differ greatly from each other^26–30^. Thus, it is very important to identify the type of programmed necrosis in a given tissue/pathology so that the correct treatment can be chosen. The results of our study have shown that not one, but four types of programmed necrosis—necroptosis, pyroptosis, oxytosis/ferroptosis, and parthanatos—are simultaneously active in the retina after IR. We have tried to identify the intersecting points of these signaling cascades. This knowledge may allow us in the future to create universal and highly effective drugs for the treatment of retinal IR.

## Results

### High-throughput gene expression profiling revealed the rapid development of ischemic pathology in the retina during the first 24 h after reperfusion

**Figure 1:**
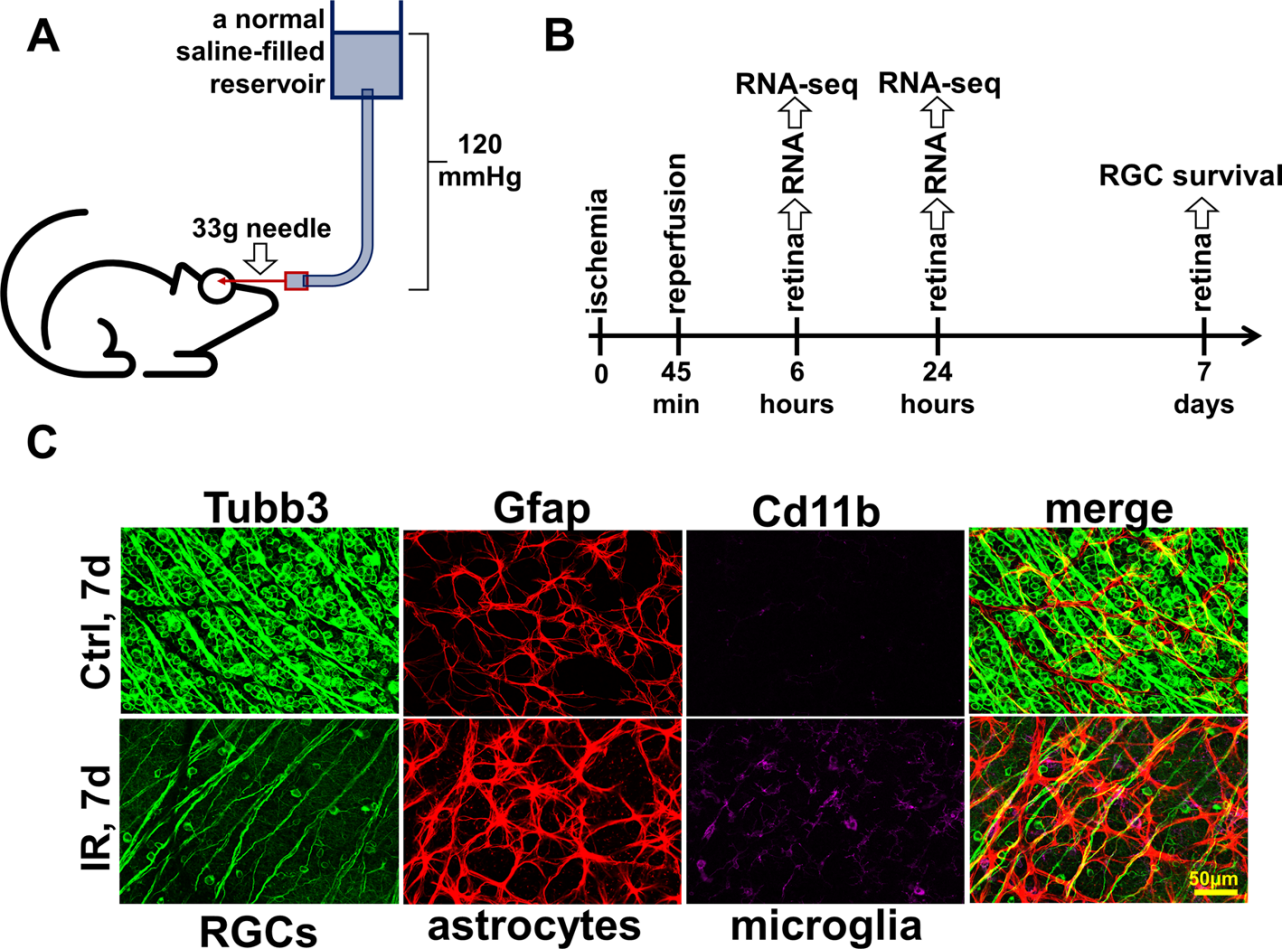
Ischemia–reperfusion (IR) leads to significant retinal damage. (**A**) Transient retinal ischemia was induced for 45 min by increasing intraocular pressure (IOP, 120 mm Hg). (**B**) Ischemic and normotensive retinas collected 6 and 24 h after reperfusion were used for RNA-seq analysis. Retinas collected 7 days after reperfusion were used to study RGC survival. (**C**) The representative confocal images indicate significant RGC death (Tubb3 as a marker) and reactive gliosis (Gfap and Cd11b as markers) in ischemic retinas 7 days after reperfusion.

**Figure 2:**
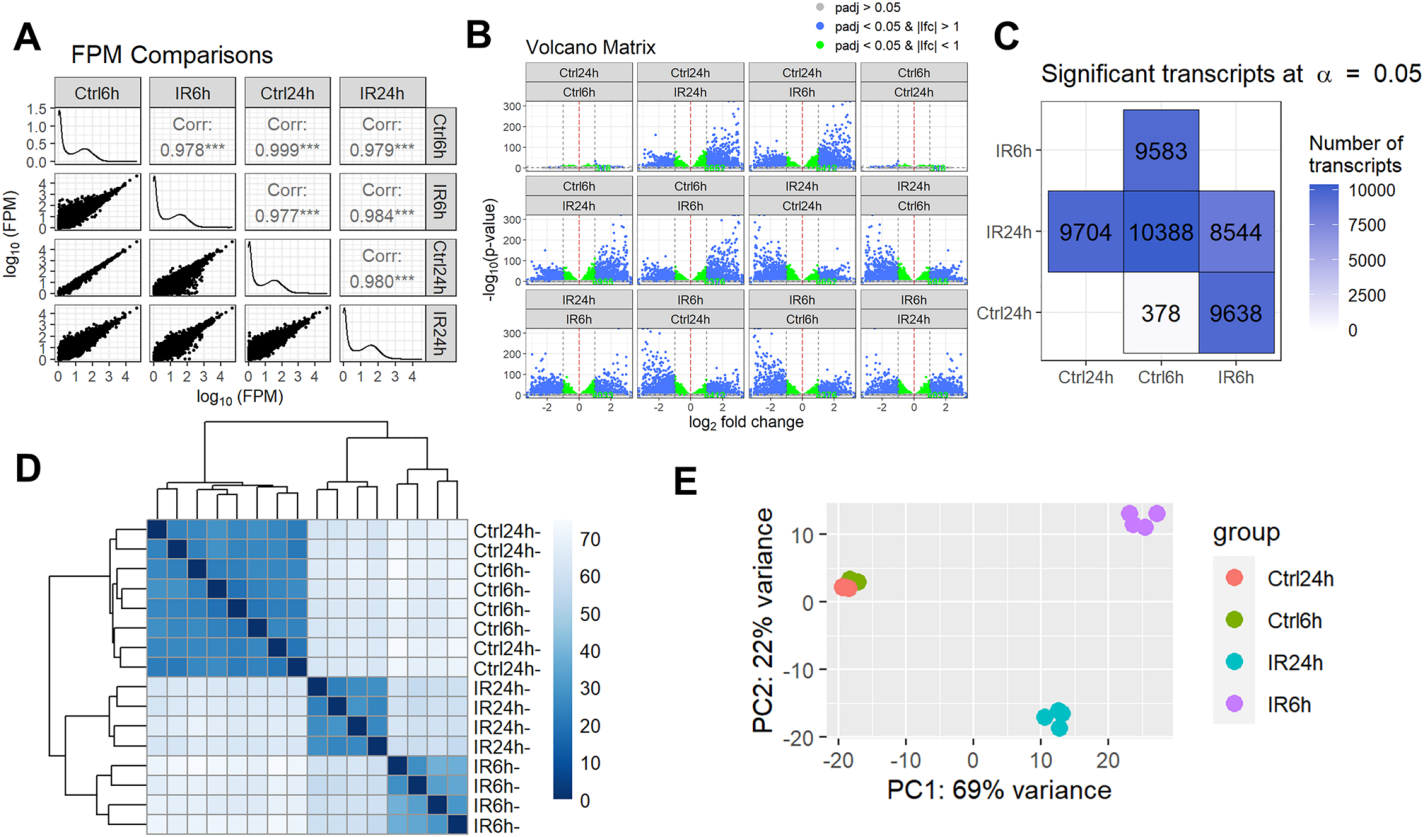
Ischemia leads to significant changes in gene expression in the retina 6 and 24 h after reperfusion. (**A**) A scatter plot matrix was used to visualize the correlation between gene expression in ischemic (IR) and normotensive (Ctrl) retinas 6 and 24 h after reperfusion. Correlation (Corr) values and FPM distributions (histograms) were generated. (**B**) A volcano plot matrix was used to combine all volcano plots for all comparisons. Each volcano plot shows the difference between two variables (either Ctrl6h, Ctrl24h, IR6h, or IR24h) in terms of gene expression values: − log10 of calculated *p* value (y-axis) is plotted against log2 fold change (x-axis). As follows from our data, the greatest difference is observed in combinations of IR6h versus Ctrl6h, IR6h versus Ctrl24h, IR24h versus Ctrl6h, IR24h versus Ctrl24h, and IR6h versus IR24h, while there is no significant difference between Ctrl6h versus Ctrl24h. (**C**) A matrix of differentially expressed genes (DEGs) at an adjusted *p* value (padj = 0.05) is consistent with the volcano plot matrix indicating that ischemia leads to a rapid change in gene expression 6 and 24 h after reperfusion. (**D**, **E**) A heatmap of the sample-to-sample distances (sample clustering, **D**) and the principal component (PCA) plot of the samples (**E**) show similar dynamics in the development of IR retinal pathology as obtained above.

The adult murine retina is more than 70% comprised of photoreceptors, while RGCs constitute less than 5% of retinal cells. The low content of RGCs in the retina poses challenges for studying changes in gene expression in these cells in response to IR stress using high-throughput approaches: highly expressed genes most likely reflect the expression of the largest cell population (photoreceptors), while low expressed genes most likely belong to the transcriptome of one of the cell populations in the retina with a small cell number like RGCs. The high-throughput microarray technology does not allow studying genes with low expression in tissue due to the strong influence of background noise. Meanwhile, the use of high throughput next generation sequencing (NGS) technology to study gene expression (RNA-seq) avoids this limitation: the presence of a transcript sequence (read/fragment) in the data is indicative of the expression of the gene in the tissue. The only limitation is the sequencing depth of the RNA-seq library, which determines the possibility of data analysis using statistical methods. To this end, transient retinal ischemia was induced for 45 min in the left eye of the 2-month-old mice (the right eye served as a normotensive control) (Fig. 1). Ischemic and control retinas were collected 6 and 24 h after reperfusion and used for preparation of RNA-seq libraries for NGS (n = 4 biological replicates; 16 RNA-seq libraries total). 50,159,699 ± 885,414 fragments (or more than 100 M reads) on average per library were sequenced among which 38,940,203 ± 808,845 fragments were uniquely mapped to the mouse genome. By analyzing these data, we found significant changes in gene expression in ischemic retinas compared to controls: the expression of 9583 genes has been changed at 6 h and the expression of 9704 genes has been changed at 24 h (Fig. 2, Supplementary Data S1). We also found a significant difference between the transcriptomes of the ischemic retinas at 6 and 24 h after reperfusion (8544 genes), while the transcriptomes of the control retinas did not differ much from each other at 6 and 24 h after reperfusion (378 genes) (Fig. 2, Supplementary Data S1). These data indicate a significant difference in the course of ischemic pathology in the retina at 6 h and 24 h after reperfusion.

**Figure 3:**
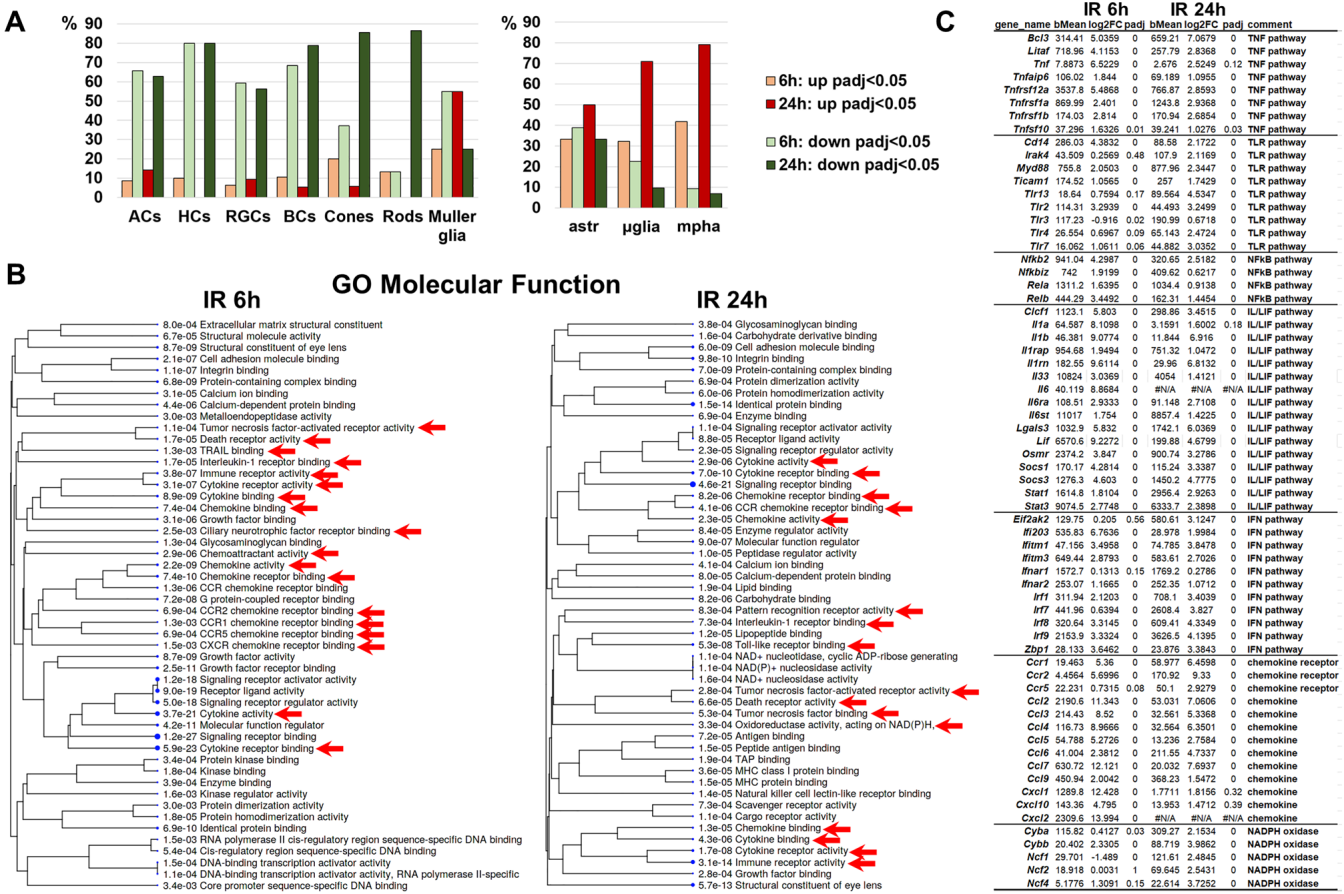
Retinal IR negatively affects the activity of genes necessary for the normal function of neurons, leads to reactive gliosis and enhances the toxic pro-inflammatory response. (**A**) The expression of a significant number of genes that are markers of retinal neurons was downregulated in the IR retina, while the expression of a significant number of glial cell and macrophage markers was increased. The number of genes with increased and decreased expression is presented as a percentage (%) of their total number. ACs—amacrine cells, HCs—horizontal cells, BCs—bipolar cells, astr—astrocyte, µglia—microglia, mpha—macrophages. (**B**, **C**) The results of the gene ontology (GO) and pathway analysis showed that many genes with increased expression in the IR retina are involved in the neurotoxic pro-inflammatory response.

The significant depth of sequencing of our RNA-seq libraries allowed us to detect the expression of not only cone and rod photoreceptor markers, but also markers of other native retinal cell types (RGCs, amacrine cells, horizontal cells, bipolar cells, and Muller glia). We found significantly reduced expression of most RGC, amacrine, horizontal, and bipolar cell markers in ischemic retinas 6 and 24 h after reperfusion (Fig. 3A, Supplementary Data S2). The expression of photoreceptor markers was less affected in ischemic retinas 6 h after reperfusion. However, the expression of almost all markers of these cells was reduced in ischemic retinas 24 h after reperfusion. The behavior of Muller glia markers was inverted: the expression of most Muller glia markers was reduced in ischemic retinas 6 h after reperfusion, while 24 h after reperfusion the expression of most Muller glia markers was increased (Fig. 3A, Supplementary Data S2). Our analysis of the expression of astrocyte markers revealed the same behavior. At the same time, the expression of many markers of microglia and macrophages was already upregulated in ischemic retinas 6 h after reperfusion; by 24 h, the number of upregulated microglia/macrophage markers reached more than 70% (Fig. 3A, Supplementary Data S2). These changes in expression reflect the processes of glial cell activation (known as reactive gliosis) and macrophage migration into the ischemic retina accompanying the inflammatory response in this tissue. Specifically, our data suggest that the activity of the TNF and IL/LIF pathways prevails in the ischemic retinas 6 h after reperfusion, while the activity of the TLR pathway predominates in the ischemic retinas 24 h after reperfusion (Fig. 3B,C, Supplementary Data S3). We found high expression of IFN pathway genes in the ischemic retina 6 and 24 h after reperfusion (Fig. 3B,C, Supplementary Data S3). Our data indicate that the genes encoding chemokines were highly expressed in the ischemic retinas 6 h after reperfusion, while the genes encoding their receptors were expressed more strongly in the ischemic retinas 24 h post-reperfusion. We also found increased expression of genes encoding NADPH oxidases, enzyme complexes responsible for the production of reactive oxygen species (ROS), in the ischemic retinas 24 h after reperfusion (Fig. 3B,C, Supplementary Data S3). All of these findings suggest that the inflammatory response changes greatly over time in the ischemic retina.

### Genes of necroptosis, pyroptosis, oxytosis/ferroptosis, and parthanatos signaling pathways are upregulated in the ischemic retina 6 and 24 h after reperfusion

**Figure 4:**
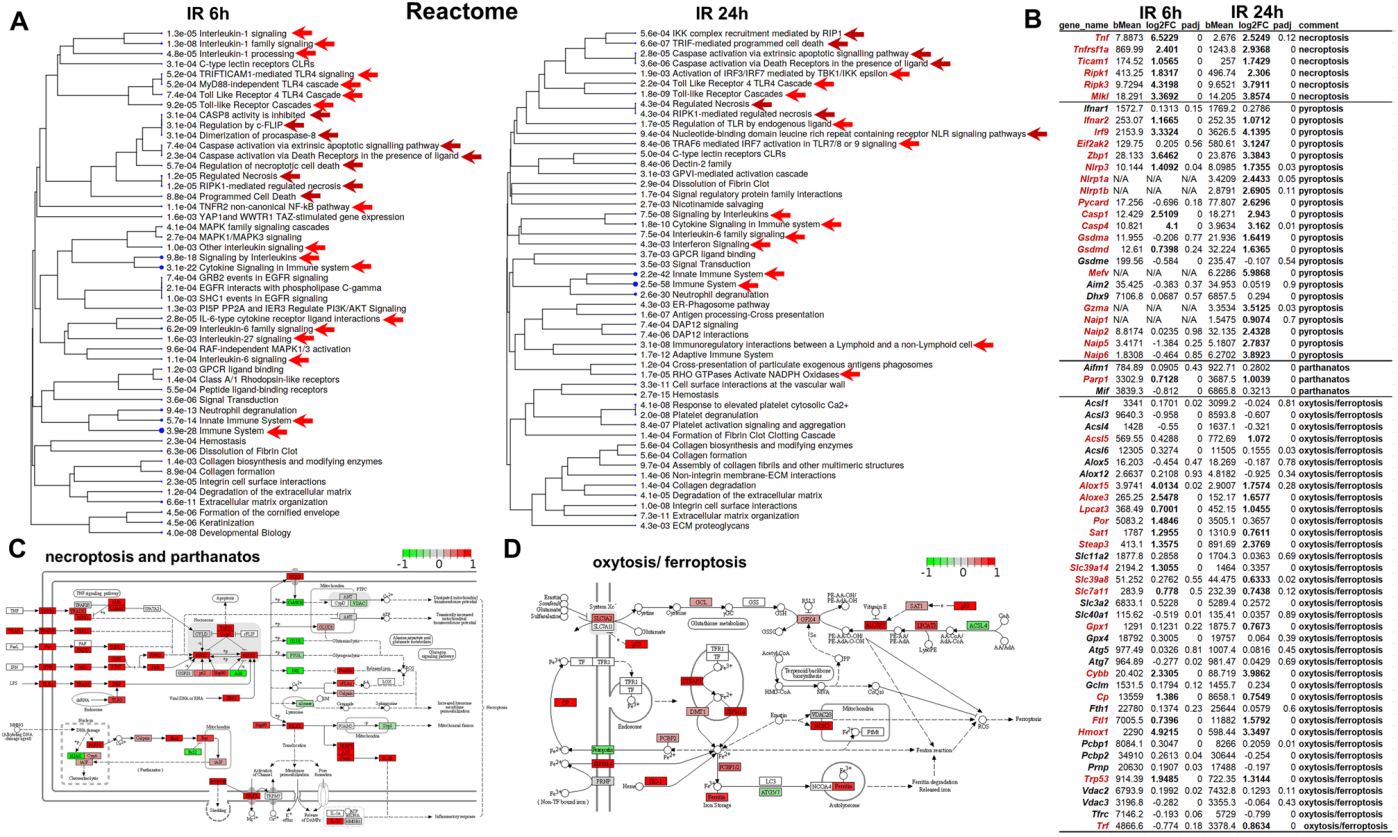
Multiple types of programmed necrosis are active simultaneously in the IR retina. (**A**) The results of the pathway analysis revealed that many genes with increased expression in the ischemic retina are involved in programmed (regulated) necrosis (red arrows indicate pro-inflammatory signaling cascades, dark red arrows indicate signaling cascades involved in programmed necrosis). (**B**) The expression of many genes regulating necroptosis, pyroptosis, oxytosis/ferroptosis, and parthanatos is significantly increased in the ischemic retina 6 and 24 h after reperfusion. We highlighted in dark red those genes whose expression was significantly increased (two-fold or more) in IR retinas. (**C**, **D**) The representative KEGG pathway maps show increased expression of necroptosis, oxytosis/ferroptosis, and parthanatos genes in the IR retina. The red box corresponds to increased gene expression in the ischemic retina, the green box corresponds to reduced gene expression in the ischemic retina.

The pathway analysis revealed that many genes involved in programmed necrosis were upregulated in the ischemic retina (Fig. 4A, Supplementary Data S3). To conduct a detailed analysis, we have compiled a list of genes belonging to signaling cascades regulating programmed necrosis such as necroptosis, pyroptosis, oxytosis/ferroptosis, and parthanatos (Fig. 4B). We found that necroptosis-pathway-associated genes (*Tnf*: log2FC 6.5 [adjusted *p* value (padj) < 0.001] 6 h IR vs. 2.5 [padj < 0.001] 24 h IR; Tnfr1*/Tnfrsf1a*: log2FC 2.4 [padj < 0.001] 6 h IR vs. 2.9 [padj < 0.001] 24 h IR; *Ripk1*: log2FC 1.8 [padj < 0.001] 6 h IR vs. 2.3 [padj < 0.001] 24 h IR; *Ripk3*: log2FC 4.3 [padj < 0.001] 6 h IR vs. 3.8 [padj < 0.001] 24 h IR; *Mlkl*: log2FC 3.4 [padj < 0.001] 6 h IR vs. 3.9 [padj < 0.001] 24 h IR) were significantly upregulated in the ischemic retinas 6 and 24 h after reperfusion (Fig. 4B). The expression of the key parthanatos pathway gene *Parp1* increased in the ischemic retina over time and reached a two-fold excess compared to the normotensive retina 24 h after reperfusion (log2FC 0.7 [padj < 0.001] 6 h IR vs. 1 [padj < 0.001] 24 h IR). Our data indicate that the expression of genes encoding proteins responsible for ferrous iron (Fe2+) production (*Steap3*: log2FC 1.4 [padj < 0.001] 6 h IR vs. 2.4 [padj < 0.001] 24 h IR) and transport (*Slc39a14*, and *Slc39a8*) was significantly increased in the ischemic retina (Fig. 4B). Meanwhile, expression of the *Slc40a1* iron transporter that is responsible for the release of Fe2+ into the extracellular space was reduced in the ischemic retinas 6 h after reperfusion (Fig. 4B). All these events may create the conditions for ferrous iron accumulation in ischemic retinal cells, leading to oxytosis/ferroptosis. We also found increased expression of *Acsl5, Alox15*, *Aloxe3*, *Lpcat3,* and *Por* genes that regulate lipid peroxidation, leading to oxytosis/ferroptosis. Our data demonstrate increased expression of genes of the gasdermin family (*Gsdma*: log2FC − 0.2 [padj < 0.001] 6 h IR vs. 1.6 [padj < 0.001] 24 h IR; *Gsdmd*: log2FC 0.7 [padj < 0.001] 6 h IR vs. 1.6 [padj < 0.001] 24 h IR) and the NLRP family (*Nlrp3*, *Nlrp1a*, and *Nlrp1b*), as well as *Casp1* (log2FC 2.5 [padj < 0.001] 6 h IR vs. 2.9 [padj < 0.001] 24 h IR), *Casp4/*Casp11 (log2FC 4 [padj < 0.001] 6 h IR vs. 3.1 [padj < 0.001] 24 h IR), and *Pycard* (log2FC − 0.7 [padj < 0.001] 6 h IR vs. 2.6 [padj < 0.001] 24 h IR) in the ischemic retinas 24 h after reperfusion (Fig. 4B). Since the activity of all these genes is required to induce pyroptosis, we would expect that many cells in the ischemic retina may undergo pyroptosis 24 h after reperfusion. Thus, our results suggest that several signaling cascades regulating programmed necrosis (necroptosis, pyroptosis, oxytosis/ferroptosis, and parthanatos) are active simultaneously in the ischemic retina (Fig. 4C,D).

### Mlkl- and gasdermin- dependent types of programmed necrosis contribute significantly to IR-induced retinal injury

**Figure 5:**
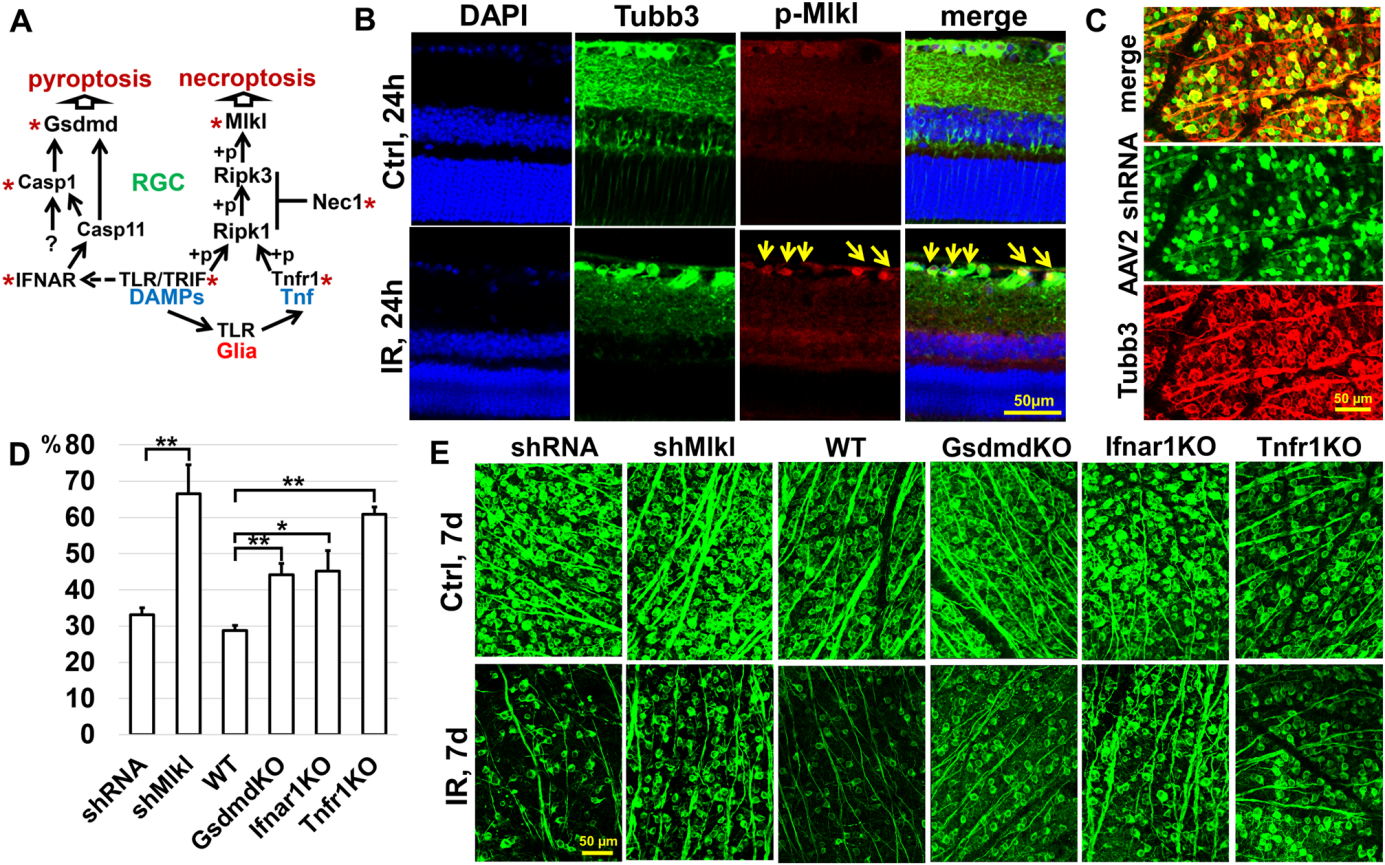
The necroptosis and pyroptosis signaling pathways are active in the IR retina, leading to significant RGC death. (**A**) Inactivation of genes of the necroptosis and pyroptosis signaling pathways significantly reduces retinal damage after IR. The star (*) indicates the genes and inhibitors tested in this and previous studies. (**B**) Immunohistochemistry (IHC) analysis indicates that the Mlkl protein is phosphorylated in RGCs of the IR retina. (p-Mlkl—phosphorylated Mlkl, Tubb3—an RGC marker) (**C**) The representative confocal images show that AAV2 exclusively transduces RGCs in the ganglion cell layer of adult animals with high efficiency. (**D**) The graph shows the percentage of surviving RGCs in ischemic retinas of animals with reduced activity of the necroptosis and pyroptosis pathways 7 days after reperfusion. (**E**) The representative confocal images show that a decrease in the *Mlkl*, *Gsdmd*, *Ifnar*, and *Tnfr1* expression in the ischemic retina leads to increased survival of RGCs 7 days after reperfusion.

Necroptosis, or Mlkl-dependent programmed necrosis, occurs after an inflammatory cytokine Tumor Necrosis Factor alpha (TNF/Tnf) or DAMPs, bind to the receptors Tnfr1/Tnfrsf1a, Tlr4, and Tlr3 (the latter two are linked to the TRIF/Ticam1 signaling cascade), causing the necrosome (primarily composed of Ripk1, Ripk3, and Mlkl proteins) to form. Ripk1, Ripk3 and Mlkl are phosphorylated during the assembly of the necrosome, which facilitates Mlkl oligomerization. Oligomerized Mlkl translocates to the plasma membrane, where it mediates increased osmotic pressure, eventually leading to membrane rupture and necrosis (Fig. 5A). Pyroptosis, also known as gasdermin-dependent programmed necrosis, depends on the caspase-mediated (Casp1 and Casp4/Casp11) release of gasdermin-N domains with their subsequent oligomerization and transmembrane pore formation followed by loss of the plasma membrane integrity and necrosis. In addition to necroptosis, the TRIF signaling cascade regulates pyroptosis, activating the IFNα/β receptor (IFNAR), which is composed of two chains: Ifnar1 and Ifnar2. Next, IFNAR promotes Casp11/Casp4 expression, which is essential for Casp1 activation (Fig. 5A). As we have noted above, the expression of all these genes is increased in the ischemic retina (Figs. 3C and 4B).

We have shown previously that Ripk1 and Ripk3 are present in normal and ischemic RGCs^31^. We found that an inhibitor of necroptosis, Necrostatin 1, a compound that prevents necrosome formation, effectively protects ischemic RGCs from death^31^. To test the role of Mlkl in RGC death after IR, we first examined the presence of the phosphorylated form of this protein in the ischemic retina 24 h after reperfusion. Our data indicate that the phosphorylated Mlkl is located in ischemic RGCs (Fig. 5B). Next, we reduced Mlkl levels in RGCs using RNA interference technology (shRNA). To deliver the shMlkl into RGCs, we used adeno-associated virus serotype 2 (AAV2) viral particles. At the very beginning of the study, we showed that our AAV2-shMlkl viral particles reduce Mlkl expression in RGCs (58% knockdown of mRNA in primary RGC cultures, *p* value < 0.05). Our data also indicate that the AAV2 vector is an effective means of delivering genetic material into adult RGCs (Fig. 5C). AAV2-shMlkl viral particles were injected intravitreally into the left eyes of anesthetized mice at least two weeks before retinal IR. Control animals were transduced with vector containing scrambled shRNA. The animals’ right eyes were used as untreated normotensive controls. Retinas were collected seven days after reperfusion then stained for the RGC marker Tubulin-βIII (Tubb3). By counting the number of Tubb3-positive cells, we determined that the number of viable RGCs was significantly higher in the AAV2-shMlkl treated retinas compared to AAV2-shRNA-treated controls (67 ± 8% vs. 33 ± 2%, *p* value < 0.01) (Fig. 5D,E).

Our data denote an increased expression of gasdermin D (*Gsdmd*) in the ischemic retina 6 and 24 h after reperfusion (Fig. 4). Pronin et al. have previously shown *Gsdmd* expression in ischemic RGCs^10^. To establish a direct link between pyroptosis/gasdermin-dependent programmed necrosis and RGC death in ischemic retinas, we used Gsdmd knockout animals (GsdmdKO). Retinal ischemia was induced in these mice and the retinas were collected 7 days after reperfusion. Whole retina flat-mounts were stained for the RGC marker Tubb3 to quantify the number of surviving RGCs in ischemic and control retinas. We found that the number of viable RGCs was significantly higher in the retinas of GsdmdKO mice compared to wild type (WT) controls (44 ± 32% vs. 29 ± 1%, *p* value < 0.002). Thus, Gsdmd deficiency in the ischemic retina improves RGC survival (Fig. 5D,E).

Finally, we assessed the contribution of Tnf/Tnfr1 and TLR/TRIF signaling cascades to retinal IR injury. We have previously shown that genetic ablation of Tlr4 and TRIF (Ticam1) results in a significant increase in RGC survival after retinal IR injury^7–9^. Here, we have evaluated the role of Tnfr1 and Ifnar1 receptors in retinal IR using Tnfr1 and Ifnar1 knockout animals (Tnfr1KO and Ifnar1KO). After retinal ischemia was induced in these animals, experimental and control retinas were collected seven days after reperfusion. We found high RGC survival in ischemic retinas of Tnfr1KO and Ifnar1KO animals compared to WT mice (Tnfr1KO: 61 ± 2%, *p* value < 0.0001; Ifnar1KO: 45 ± 6%, *p* value < 0.02; WT: 29 ± 1%). Since Tnf/Tnfr1 and TLR/TRIF signaling cascades are responsible not only for necroptosis and pyroptosis, but also regulate the inflammatory response in the tissue, it is difficult to separate the contribution of these cascades to necrosis and inflammation in the ischemic retina. However, this is not necessary since programmed necrosis and inflammation are interconnected by activating each other through the positive feedback described in the introduction. Thus, programmed necrosis and inflammation are parts of a gene regulatory network (GRN), and Tnf/Tnfr1 and TLR/TRIF cascades are important parts of it.

### Ferrous iron (Fe2+), reactive oxygen and nitrogen species-dependent types of programmed necrosis play an essential role in the IR retina

**Figure 6:**
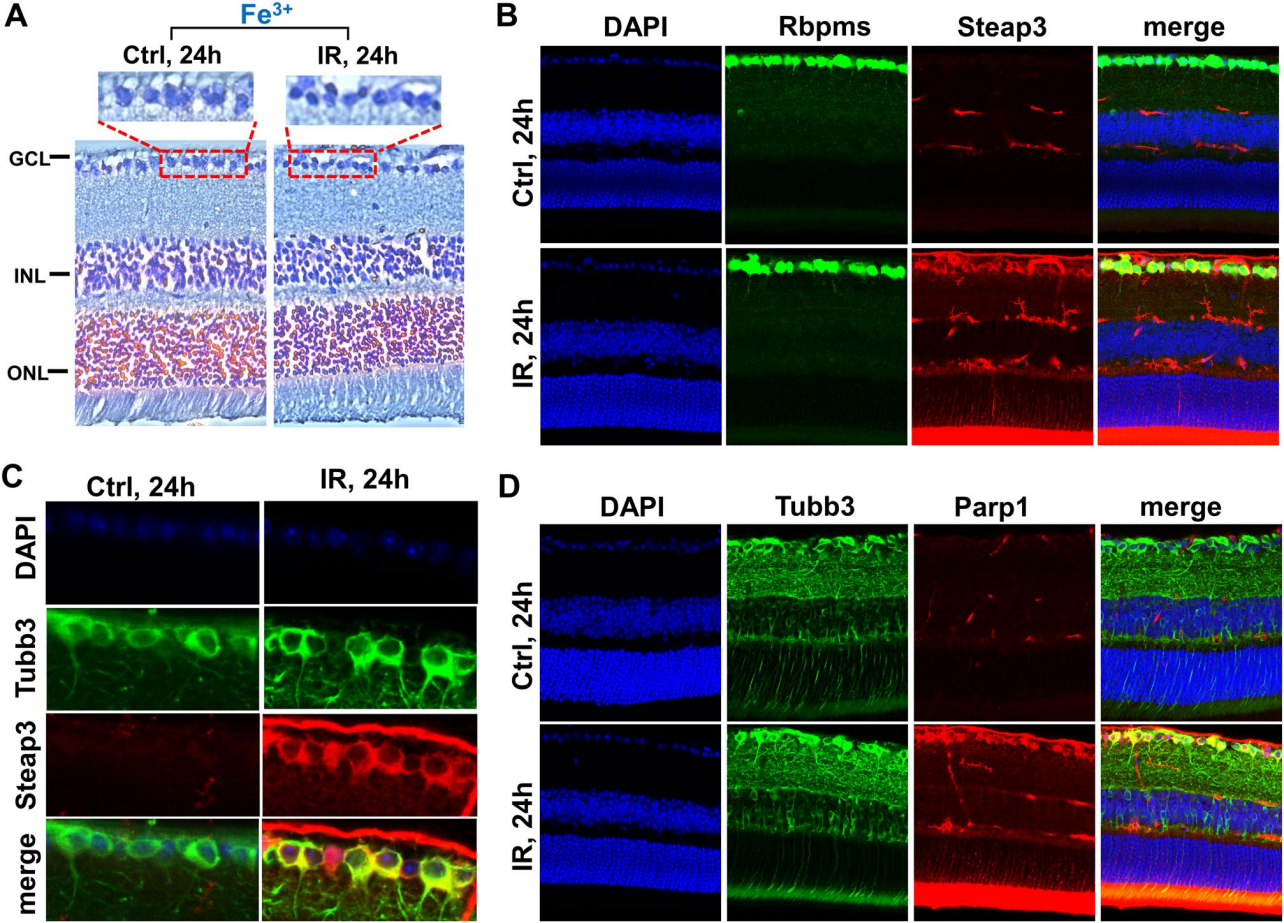
The expression of genes involved in oxytosis/ferroptosis, and parthanatos is significantly increased in ischemic RGCs. (**A**) Prussian blue stain indicates a high content of iron in neurons of ischemic and normotensive retinas. Prussian blue is specific for ferric iron (Fe3+). (**B**, **C**) Immunohistochemistry (IHC) analysis reveals that Steap3, the key enzyme generating ferrous (Fe2+) iron from ferric (Fe3+) iron, is highly expressed in the ischemic retina. Rbpms and Tubb3 are RGC markers. (**D**) IR results in high expression of Parp1 in RGCs.

Reactive oxygen and nitrogen species (ROS/RNS), such as the hydroxyl radical, peroxynitrite, hydroperoxyl radical, and carbonate radical anion, are generated from the superoxide, nitric oxide (NO), hydrogen peroxide, and carbon dioxide in the Fenton/Haber–Weiss reaction that is only possible in the presence of ferrous iron (Fe2+) as a catalyst^43,44^. High hydroxyl radical, peroxynitrite, hydroperoxyl radical, carbonate radical anion levels cause significant oxidative DNA and membrane damage. An iron is a transition metal and exists in living cells as ferric (Fe3+, the stable form of iron) and ferrous (Fe2+) ions. Retinal cells contain large amounts of iron, which is vital for retinal metabolism^45–47^. Our data suggest a high content of ferric iron (Fe3+) in ischemic and normotensive RGCs (Fig. 6A). Ferrous iron (Fe2+) is predominantly found in the catalytic centers of enzymes and is very dangerous in its free form, because of its ability to catalyze the Fenton/Haber–Weiss reaction. Steap3 is the key enzyme capable of generating ferrous (Fe2+) iron from ferric (Fe3+) iron^48^. Our data indicate an almost threefold and more than fivefold excess of *Steap3* expression in the ischemic retina compared to the control 6 and 24 h after reperfusion, respectively (log2FC 1.4 [padj < 0.001] 6 h IR vs. 2.4 [padj < 0.001] 24 h IR). Our data demonstrate the predominant localization of Steap3 in RGCs, and its expression is significantly higher in ischemic RGCs compared to control RGCs 24 h after reperfusion (Fig. 6B,C). Thus, a large amount of the Steap3 enzymes in ischemic RGCs may lead to the generation of large amounts of free ferrous (Fe2+) iron, resulting in significant oxidative DNA and membrane damage. Significant oxidative DNA damage promotes Parp1 overactivation, leading to excessive poly(ADP-ribose) (PAR) production and its binding to apoptosis-inducing factor (AIF/Aifm1) located in the outer mitochondrial membrane. These events promote AIF release into the cytosol, where it binds to macrophage migration inhibitory factor (Mif). Together, they penetrate the cell nucleus where Mif degrades genomic DNA into 20–50 kb DNA fragments, leading to necrotic cell death known as parthanatos^29^. Our data indicate an increased expression of Parp1 (log2FC 0.7 [padj < 0.001] 6 h IR vs. 1 [padj < 0.001] 24 h IR) in ischemic RGCs (Fig. 6D). A significant increase in PAR levels in the ganglion cell and the inner nuclear layers of the ischemic retina has also been shown at 12 and 18 h after reperfusion^40^. The role of parthanatos in retinal IR injury and RGC death was shown previously using Parp1 and Aifm1 inhibitors ^38–42^. Meanwhile, the plasma membrane of retinal neurons is more susceptible to oxidative damage compared to other mammalian tissues since they are highly enriched in polyunsaturated fatty acids (PUFAs)^49^. Unsaturated bonds are more prone to free radical attack, leading to lipid peroxidation followed by plasma membrane damage and necrotic cell death. This process is known as oxytosis/ferroptosis since iron plays a significant role^28,48^. The accumulation of lipid peroxides formed by free radicals has been shown in the ischemic retina^50,51^. The role of oxytosis/ferroptosis in retinal IR injury and RGC death was conveyed previously using numerous inhibitors and iron-chelating agents^32,34–37^. Since oxytosis/ferroptosis as a type of programmed necrosis was described only after 2013 and many of these referenced articles appeared before 2013, we are only now understanding the significance of these studies. Since parthanatos and oxytosis/ferroptosis are dependent on the presence of ferrous (Fe2+) iron and their role has already been shown in the ischemic retina, the ferrous (Fe2+) iron-generating Steap3 enzyme may be key in the regulation of parthanatos and oxytosis/ferroptosis in the ischemic retina.

### Cross talk between programmed necrosis signaling pathways in the IR retina

**Figure 7:**
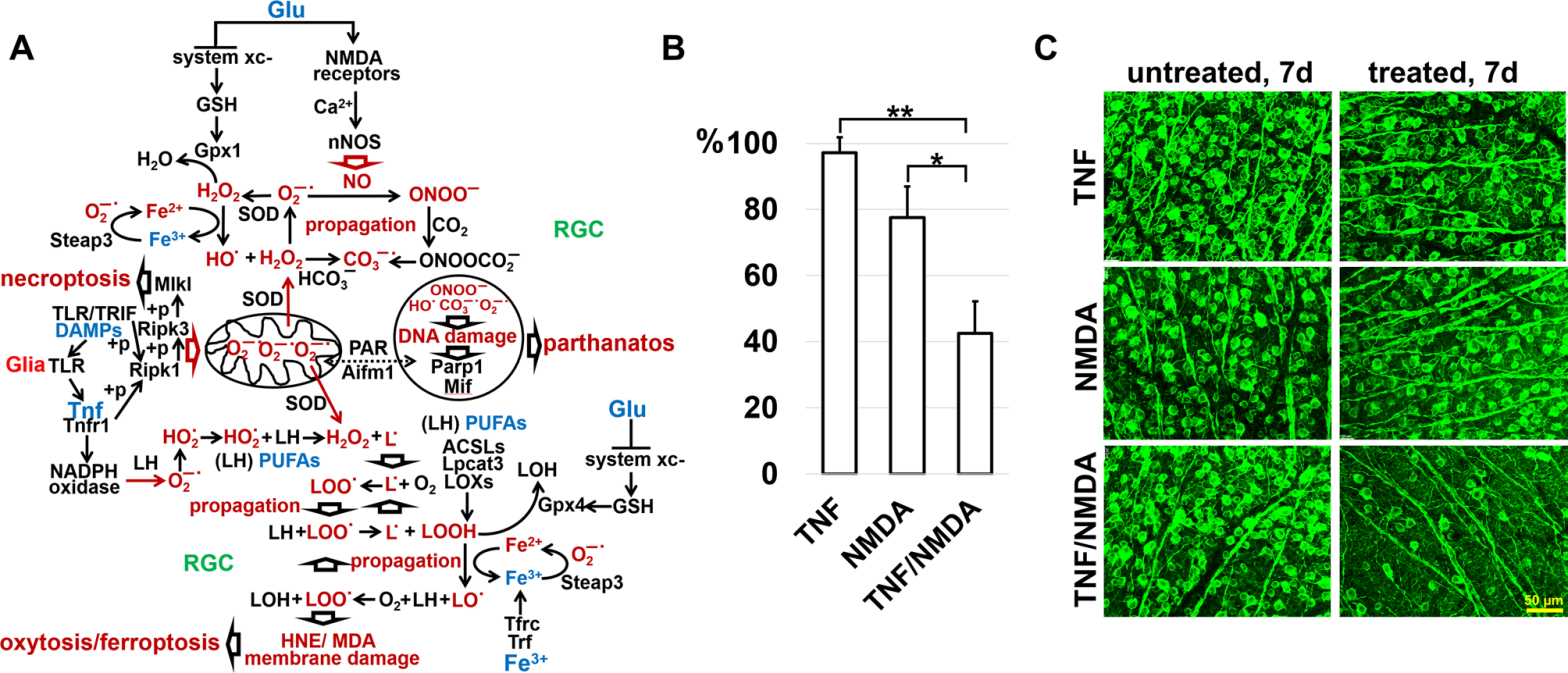
Three compounds, Tnf, glutamate, and iron, present at high concentrations in the IR retina, are likely to promote at least necroptosis, oxytosis/ferroptosis, and parthanatos, leading to significant RGC death. (**A**) The necroptosis, oxytosis/ferroptosis, and parthanatos signaling cascades are interconnected with each other and, in the presence of Tnf, glutamate, and iron, they can be started simultaneously with high efficiency. (**B**, **C**) Since iron is already present in RGCs, intravitreal injection of both Tnf (TNF) and glutamate (NMDA) is sufficient to cause significant damage to the naïve retina.

The activation of four types of programmed necrosis—necroptosis, pyroptosis, oxytosis/ ferroptosis, and parthanatos—in the IR retina poses serious challenges: we now have to create drugs that will be efficient against not one but four types of programmed necrosis. To this end, based on the data obtained here and earlier, we should build a gene regulatory network (GRN) that will include necroptosis, pyroptosis, oxytosis/ferroptosis, and parthanatos signaling pathways. Pyroptosis could occur through the following signaling cascade: DAMPs-TLR/TRIF-IFNAR-Casp11/Casp4-Casp1-Gsdmd (Fig. 5A). The problem arises because the rates of survival of ischemic RGCs do not differ between Casp11/Casp4 deficient and wild type mice^10^. Thus, either Casp11/Casp4 is not involved in RGC pyroptosis, or a new approach is needed to study the role of Casp11/Casp4 in RGC pyroptosis. At the same time, we managed to build the GRN that includes necroptosis, oxytosis/ferroptosis, and parthanatos (Fig. 7A). The initial IR stress leads to the accidental (uncontrolled) necrotic retinal cell death followed by accumulation of high levels of extracellular DAMPs, which activate glial toll-like receptors (TLRs), leading to Tnf production (our data supports this sequence of events). Tnf via Tnfr1 and DAMPs via TLR/TRIF trigger Ripk1/Ripk3/Mlkl autophosphorylation followed by necroptosis in some ischemic RGCs. Simultaneously, the Tnf pathway promotes production of ROS in those RGCs that have survived: (1) the translocation of phosphorylated Ripk1/Ripk3/Mlkl to the mitochondrial membrane stimulates the production of mtROS; (2) Tnf-dependent NADPH oxidase activity leads to an increased level of ROS in close proximity to the plasma membrane since these enzymes are located on it^52–54^. We found in our study increased expression of genes encoding NADPH oxidases in the IR retina. We have also previously shown the important role of NADPH oxidases in ischemic RGCs^55,56^. It should be noted that over the past 30 years, a huge amount of evidence has accumulated indicating the critical role of ROS in IR-induced RGC death and retinal degeneration^1,55–60^. Since the retina contains a large number of excitatory types of retinal neurons whose main neurotransmitter is glutamate, the necrotic death of these neurons should be also accompanied by the release of a significant amount of glutamate. Thus, it is not surprising that a significant release of glutamate during retinal ischemia and after reperfusion has been previously observed^61,62^. The opening of NMDA-dependent ion channels by glutamate triggers a rapid influx of calcium into neurons, leading to the neuronal nitric oxide synthase (nNOS) activation and production of nitric oxide (NO). The large quantity of extracellular glutamate also inhibits glutathione production, the neuronal major antioxidant, leading to elevated ROS levels. Increased Steap3 activity, resulting in high ferrous (Fe2+) iron content, raised levels of ROS and NO may start an uncontrolled production of reactive oxygen and nitrogen species via the Fenton/Haber–Weiss reaction in ischemic RGCs leading to lipid peroxidation, and DNA damage and resulting in oxytosis/ferroptosis, and parthanatos, respectively. One, two, or all of these types of programmed necrosis activated in a single RGC can lead to a loss of membrane integrity and the release of DAMPs and glutamate into the extracellular space, repeating this dangerous process and causing significant retinal damage. Since glutamate and Tnf could start the described process, and iron is already present in RGCs, we injected into the vitreous of the naïve mice (1) TNF (0.5 ng/µl, 2 µl; n = 4), (2) NMDA as a glutamate analog (10 mM, 2 µl; n = 6), and (3) TNF and NMDA (n = 4). We selected such concentrations of TNF and NMDA that, injected separately, they did not lead to significant RGC death (Fig. 7B,C). TNF and NMDA were injected into the left eyes of the animals. The right eyes were saved as untreated controls. RGC survival in these animals was determined after 7 days. We found that the administration of both compounds resulted in retinal damage similar to the damage that occurs after IR, while individually, TNF and NMDA have caused minor damage (TNF: 97 ± 5%, *p* value < 0.003; NMDA: 77 ± 9%, *p* value < 0.03; TNF/NMDA: 42 ± 10%; Fig. 7B,C). Thus, DAMPs/Tnf, glutamate, and iron may be key players simultaneously triggering several types of programmed necrosis in ischemic RGCs (Fig. 7).

## Discussion

Retinal IR is an important risk factor in numerous optic neuropathies^1–6^. Sterile inflammation is the key player in the pathogenesis of many of these ocular diseases and necrotic cell death is the factor that triggers and maintains this neurotoxic process in the retina^7–42^. Necrotic cell death can be either accidental or programmed^26–30^. Since accidental necrosis occurs after strong stresses and cannot sustain itself, it is only important as a trigger for sterile inflammation. Meanwhile, programmed necrosis and sterile inflammation, acting as parts of a positive feedback loop, can maintain each other, leading to severe tissue damage (secondary injury). There are many types of programmed necrosis that differ in their signaling cascades, leading to loss of plasma membrane integrity, a hallmark of necrotic cell death^26–30^. The objective of this study was to investigate what type of programmed necrosis contributes to retinal damage after IR, using a variety of approaches including high-throughput expression profiling. We found that not one, but at least four types of programmed necrosis including necroptosis, pyroptosis, oxytosis/ferroptosis, and parthanatos are active simultaneously in the IR retina. Our data suggest that the high activity of the Tnf signaling cascade at the early stage of retinal ischemia leads to the predominant activation of necroptosis 6 h after reperfusion, while other types of programmed necrosis are more active 24 h after reperfusion. Our data suggest that DAMPs/Tnf, glutamate, and ferrous (Fe2+) iron generated by Steap3 may be key players concurrently triggering necroptosis, oxytosis/ferroptosis, and parthanatos in ischemic RGCs. The mechanism triggering pyroptosis in the ischemic retina is not completely clear.

The types of programmed necrosis active in the IR retina could be divided into Mlkl/gasdermin—dependent (necroptosis and pyroptosis) and Fe2+/ROS/RNS-dependent (oxytosis/ferroptosis, and parthanatos). This means that Tnf/TRIF-dependent Ripk1/Ripk3/Mlkl phosphorylation or caspase-mediated release of gasdermin-N domains can trigger necroptosis or pyroptosis, respectively, regardless of the presence or absence of reactive oxygen and nitrogen species (ROS/RNS). Our findings here and previously published data indicate that necroptosis and pyroptosis make a significant contribution to IR-induced retinal damage^10,31–33,63^. However, necroptosis and pyroptosis are likely extreme events resulting from excessive Ripk1/Ripk3/Mlkl phosphorylation and release of gasdermin-N domains. It has been reported that gasdermin-N can form transmembrane pores in the mitochondrial membrane leading to the cytochrome C release followed by apoptosis^64^. Upon Tnf signaling activation, phosphorylated Ripk1, Ripk3, and Mlkl have all been shown to translocate to the mitochondrial membrane where they may also participate in the release of cytochrome C^52,54^. We might here speculate that gasdermins and phosphorylated Ripk1/Ripk3/Mlkl connect the extrinsic (receptor-mediated) apoptotic pathway with the intrinsic (mitochondria-mediated) apoptotic pathway. However, excessive Ripk1/Ripk3/Mlkl phosphorylation and release of gasdermin-N domains may lead to uncontrolled pore formation in the mitochondrial and plasma membrane. Excessive disruption of the mitochondrial membrane integrity can lead to a loss of the mitochondrial transmembrane potential and failure of oxidative phosphorylation, resulting in the production of significant amounts of mitochondrial ROS (mtROS)^65^. It has already been shown that Tnf-dependent phosphorylation of Ripk1/Ripk3/Mlkl leads to increased mtROS production, which contributes to necroptosis^52,54^. Meanwhile, uncontrolled pore formation in the plasma membrane disrupts osmotic potential and causes cell swelling followed by loss of membrane integrity and necroptosis/pyroptosis. Thus, cells in which necroptosis and pyroptosis signaling cascades were triggered but this triggering did not lead to their death, could die by oxytosis/ferroptosis or parthanatos because of high ROS production.

Our data suggest that the Tnf signaling cascade is very active in the IR retina and its inactivation leads to a high level of RGC survival. Tnf signaling has the unique ability to not only stimulate mtROS production (via Ripk1/Ripk3/Mlkl) but also to activate ROS-producing NADPH oxidases^52,53^. We found in this study increased expression of genes encoding NADPH oxidases in the IR retina. It is important to note that the NADPH oxidases are located in the plasma membrane and, thus, may directly start lipid peroxidation through ROS production (Fig. 7A). The activity of the NADPH oxidases may also affect plasma membrane lipid peroxidation of neighboring cells, provided that the membranes of such cells are located very close to each other, which is typical for RGCs and astrocytes. We have previously found that the activity of the NADPH oxidases in astrocytes and RGCs leads to oxidative stress and significant retinal damage after IR^55,56^. Since NADPH oxidases play an important role in oxytosis/ferroptosis by promotion of ROS production, Tnf signaling, thus, could be responsible not only for necroptosis, but also for oxytosis/ferroptosis in the ischemic retina^66^. Tnf signaling could also contribute to parthanatos by stimulating the production of high levels of mtROS.

Oxytosis/ferroptosis and parthanatos signaling cascades require the presence of not only ROS but also RNS and ferrous (Fe2+) iron (as a catalyst of the Fenton/Haber–Weiss reaction) in a cell^28,29,43,44,48,66^. To ensure oxytosis/ferroptosis and parthanatos, it is also necessary to reduce the level of glutathione, the main antioxidant in the cell^28,29,43,44,48,66^. It should be noted that mtROS can oxidize Ripk1 and promote Ripk1 autophosphorylation required for Ripk3/Mlkl phosphorylation^54^. Since, in turn, phosphorylated Ripk1, Ripk3, and Mlkl can stimulate the production of mtROS, this positive feedback loop is sustained, leading to the necroptosis^54^. It follows that, while Tnf signaling would trigger necroptosis, the ROS could maintain necroptosis in the cell. Thus, any compounds capable of triggering ROS/RNS production and lowering the antioxidant response could promote necroptosis, oxytosis/ferroptosis, and parthanatos in the cell. Tnf only partially meets these conditions. Meanwhile, high glutamate levels in the extracellular space, that occur after retinal IR, lead to the enhanced calcium (Ca2 +) entry into RGCs due to glutamate-dependent NMDA receptor activity^61,62^. An increase in the intracellular Ca2 + level leads to a disruption of the electrochemical gradient across the mitochondrial transmembrane, resulting in an elevation in mitochondrial superoxide levels^67^. An influx of Ca2 + into neurons also lead to the neuronal nitric oxide synthase (nNOS) activation and production of nitric oxide (NO)^68^. High extracellular glutamate levels prevent intracellular production of glutathione by inhibiting the cystine/glutamate exchanger (xCT), leading to a decreased antioxidant response^69^. We would like to point out that iron is an important component in glutamate-induced neurotoxicity. Sakamoto et al. demonstrated that iron-chelating agents reduced oxidative stress in the NMDA treated retina, resulting in significant RGC survival^70^. It's interesting that glutamate-dependent activation of nNOS can trigger a signaling cascade, which promotes iron (ferrous iron; Fe2+) uptake into the cell from the extracellular space^71^. Since iron is vital for retinal metabolism, the release of the iron-binding proteins from necrotic cells followed by denaturation of these proteins may result in the formation of a large pool of free ferrous iron in the extracellular space of IR retinas^45–47^. However, the most important source of ferrous iron (Fe2+) in the cell is Steap3, the enzyme that reduces ferric iron (Fe3+) to ferrous iron (Fe2+)^48^. Our data indicate that the *Steap3* expression is increased significantly in ischemic RGCs. Thus, Tnf, glutamate, and Steap3-generated ferrous iron (Fe2+) could simultaneously trigger and maintain several types of programmed necrosis in the IR retina (Fig. 7).

In conclusion, it should be emphasized that not one, but many types of programmed necrosis should be simultaneously targeted for therapeutic purposes in the IR retina. The success of such concurrent therapy for necroptosis and oxytosis/ferroptosis in the IR retina has already been demonstrated in Qin et al. study^32^. Since the simultaneous presence of ROS, RNS, and ferrous iron (Steap3-generated Fe2+) in the cell act as an engine of the pathological process, therapeutic interventions to reduce their levels in ischemic RGCs may be the most efficient. As Tnf/DAMPs, glutamate, iron, and their signaling cascades affect ROS, RNS, and Fe2+ levels within the cell, they could be prime targets.

## Methods

### Animals and ethics statement

All procedures were performed in compliance with the National Institutes of Health (NIH) Guide for the Care and Use of Laboratory Animals and according to the University of Miami Institutional Animal Care and Use Committee (IACUC) approved protocols. Tnfr1, Ifnar1, and Gsdmd knockout animals (Tnfr1KO, Ifnar1KO, and GsdmdKO, respectively) and C57BL/6 J mice as the wild type controls were obtained from the Jackson Laboratory (Bar Harbor, Maine, United States; stock numbers 003242, 028288, 032663, 000664). Mice were housed under standard conditions of temperature and humidity, with a 12-h light to dark cycle and free access to food and water. All animals have the C57BL/6J genetic background. All methods were completed and reported in accordance with ARRIVE guidelines.

### Transient retinal ischemia

Before anesthesia, pupils were dilated with 1% tropicamide–2.5% phenylephrine hydrochloride (NutraMax Products, Inc., Gloucester, US), and corneal analgesia was achieved with 1 drop of 0.5% proparacaine HCl (Bausch and Lomb Pharmaceuticals, Tampa, FL). To minimize pain, distress, and injury, animals were anesthetized with isoflurane for 45 min. The anesthetic was administered to the breathing mice via a nose cone. Transient retinal ischemia was induced by introducing into the anterior chamber of the left eye a 33-gauge needle attached to a saline-filled (0.9% NaCl) reservoir raised above the animal to increase intraocular pressure (IOP, 120 mm Hg). We observed a blanching of the retinal arteries and whitening of the anterior segment of the eye by microscopic examination, which is a criterion for complete retinal ischemia. The right eye was saved as a normotensive control. Body temperature was held at 37 ± 0.5 °C with a temperature-controlled heating pad. After the needle was removed, 0.5% erythromycin ophthalmic ointment (Fera Pharmaceuticals, Locust Valley, US) was applied to the conjunctival sac to prevent infection. Mice were euthanized in accordance with the recommendations of the Panel on Euthanasia of the American Veterinary Medical Association (AVMA).

### RNA extraction and quality control

Total RNA was extracted from ischemic and normotensive retinas using RNeasy Plus Mini Kit (74134, Germany), according to the manufacturer's protocol. The quality and quantity of RNA was determined using Qubit 4 Fluorometer and NanoDrop Spectrophotometer (both from ThermoFisher Scientific, US). RNA integrity was determined using 2100 Bioanalyzer Instrument (Agilent Technologies, US). The RIN score was closer to 9 or higher for all of our samples.

### Preparation of RNA-seq libraries and sequencing

Illumina Stranded mRNA Prep kit (20040532, Illumina, US) and IDT® for Illumina® RNA UD Indexes Set A (20040553, Illumina, US) were used for preparation of sequencing libraries. Briefly, 100 ng of total RNA were used to capture mRNA, which, then, were fragmented and primed for cDNA synthesis. After the first and second cDNA strands were synthesized, pre-index anchors were ligated to the ends of the double-stranded cDNA and then the dual-index adapter sequences were added by PCR amplification. The concentration and quality of the final libraries were assessed using Qubit 4 Fluorometer (ThermoFisher Scientific, US) and 2100 Bioanalyzer Instrument (Agilent Technologies, US). The sequencing libraries were multiplexed and the fragments in libraries were sequenced from both ends on the Illumina Novaseq 6000 with a 2 × 150 paired end (PE) configuration. The next-generation sequencing (NGS) was conducted at the University of Michigan Advanced Genomics Core.

### RNA-seq data analysis

Paired-end reads were aligned using basic STAR workflow. First, we generated genome indexes files using the reference genome sequences (GRCm39 FASTA files: GRCm39.primary_assembly.genome.fa) and annotations (GRCm39 GTF files: gencode.vM28.primary_assembly.annotation.gtf). Second, the reads (sequences) in the form of FASTQ files were mapped to the mouse genome. Paired read counts were quantified using HTseq and differential gene expression analysis was performed using DESeq2. Gene ontology (GO) analysis and pathway analysis were performed using ShinyGO 0.76 (http://bioinformatics.sdstate.edu/go/) and Pathview (https://pathview.uncc.edu/)^72–74^. We used ViDGER (Visualization of Differential Gene Expression Results using R) for visualizations of RNA-seq data.

### Immunohistochemistry of flat-mounted retinas and counting of ganglion cell layer neurons

Experimental and control eyes of animals were enucleated, fixed with 4% paraformaldehyde in phosphate-buffered saline (PBS, pH 7.4) solution for 1 h and were then transferred to PBS. The retinas were removed, washed three times with PBS, permeabilized with 0.5% Triton X-100 in PBS for 1 h, blocked with 0.5% Triton X-100 containing 10% donkey (or goat) serum in PBS for 1 h, and then incubated overnight in 0.2% Triton X-100/10% donkey (or goat) serum in PBS containing either beta III Tubulin antibody (Tubb3, 1:250; 802001, BioLegend, US), Cy3-conjugated anti-glial fibrillary acidic protein (GFAP) antibody (1:150; MAB3402C3, MilliporeSigma, US), or Cy5-conjugated anti-Cd11b antibody (1:100; RM2805, ThermoFisher Scientific, US). The next day, the retinas were washed with PBS three times, and goat anti-mouse AlexaFluor or donkey anti-rabbit secondary antibody (1:500; both from ThermoFisher Scientific, US) in 0.15% Tween 20/PBS was applied for 1.5 h at room temperature. After washing three times with 0.15% Tween 20/PBS, retinas were flatmounted (RGC layer facing up) and coverslipped, and imaged with a Leica STELLARIS confocal microscope (Leica Microsystems, US). Negative controls were incubated with secondary antibody only. Tubb3-positive neurons in the ganglion cell layer were imaged randomly to collect images from four retinal quadrants, with a × 20 objective lens. Tubb3-positive neurons were counted with ImageJ software. Cell loss in the ischemic retinas was calculated as a percentage of the mean cell density in normotensive fellow control eyes.

### Immunohistochemistry

Eyes were enucleated, fixed with 4% paraformaldehyde in PBS, washed with PBS, and then retinas were removed. The fixed retinas were sectioned with a vibratome (Leica Microsystems,US) to a thickness of 100 μm, and, then, sections were permeabilized with 0.3% Triton X-100/PBS for 1 h, washed three times with PBS, blocked in a buffer (5% donkey serum, 2% bovine serum albumin and 0.15% Tween-20 in PBS) for 1 h, and incubated overnight with Mlkl (phospho S345) antibody (p-Mlkl, 1:300, ab196436, Abcam, US), Steap3 antibody (1:100, pHyde, sc-376327, Santa Cruz Biotechnology, US), Parp1 antibody (1:100, sc-74470, Santa Cruz Biotechnology, US), Rbpms antibody (1:400, GTX118619, GeneTex, US), and Tubb3 antibody. The next day, the retinas were washed three times with PBS, and incubated with species-specific secondary fluorescent antibodies (ThermoFisher Scientific, US). Control sections were incubated without primary antibodies. Imaging was performed with Leica STELLARIS confocal microscope (Leica Microsystems, US).

### AAV2-shMlkl viral particles and their efficacy

The design and production of AAV2-shMlkl and scrambled viral particles was carried out by the VectorBuilder Inc company. The most potent and specific shRNA against Mlkl was used (mMlkl[shRNA#2]: AATTCGATTCTCCCAACATCTTGCGTATATTTGGGATTTGC; knockdown score—15). All vector concentrations were > 10^12^ genome copies/ml. To show that AAV2-shMlkl viral particles effectively reduce the Mlk1 levels in the RGCs, we used primary RGC cultures isolated from retinas using the two-step immunopanning protocol as described in our articles^7,8,31^. To transduce RGCs with AAV2, primary neurons were plated in 24-well plates at a density of 200,000 cells per well and viral particles (5 µl) were added to the media after 1–1.5 h. RGCs were cultured in the serum-free media (Neurobasal/B27 media; ThermoFisher Scientific, US) for the next 24 h. The serum-free media was then replaced with fresh one. AAV2-treated RGCs were collected 2 days later and used in the quantitative RT-PCR. To this end, we used Mlkl-specific primers (5ʹ–ACCCTGAAGCAATGCTCACT–3ʹ, 5ʹ–TGATCAATGCAAATCCCA–3ʹ). Relative expression was calculated by comparison with a standard curve, following normalization to the housekeeping gene 18S ribosomal RNA (Rn18s) expression (5ʹ–CGGCTACCACATCCAAGGAA–3ʹ, 5ʹ–GCTGGAATTACCGCGGCT–3ʹ).

### Intravitreal injections of AAV2 viral particles, TNF and NMDA

Animals were anaesthetized by intraperitoneal injection of ketamine (80 mg/kg)/xylazine (10 mg/kg). Intravitreal injections were performed under a microsurgical microscope using glass pipettes with a diameter of approximately 150 µm at the tip. Each eye was punctured at the upper nasal limbus and a volume of 2 μl of the viral particles (AAV2-shMlkl or scrambled viral particles), TNF (0.5 ng/µl in PBS, RMTNFAI, ThermoFisher Scientific, US), NMDA (10 mM in PBS, M3262, MilliporeSigma, US), or TNF/NMDA was injected. To allow diffusion of the solution, the pipette was kept in place for about 15 s.

### Prussian blue stain

To detect ferric iron (Fe3+) in the control and ischemic retinas, we used Iron Stain Kit (ab150674, Abcam, US) according to the manufacturer's protocol.

### Statistical analysis

All of our experiments used power analysis to determine the appropriate number of experiments. All data for hypothesis testing were examined for Gaussian distribution prior to further statistical analysis. The unpaired Student’s t-test was used for experiments containing one variable. *p* values equal to or less than 0.05 were considered statistically significant. For experiments containing two or more variables, a one-way analysis of variance (ANOVA) was used with the appropriate multiple comparison post-hoc test (such as Newman-Keuls or Dunns). Protocols using a range of genotypes or drug treatments were designed with individual treatments being assigned in a random fashion. Treatments were assigned blindly to the experimenter by another individual in the lab. Negative and positive controls were used in our study. Generation and analysis of next-generation sequencing (NGS) data were conducted in-house according to ENCODE standards and pipelines with n = 4 for RNA-seq data.

## Supplementary Information

Dataset S1.

Dataset S2.

Dataset S3.

## Data Availability

The datasets obtained in this study are available in the BioProject database, accession number PRJNA859197.
